# Structure of Actin-related protein 8 and its contribution to nucleosome binding

**DOI:** 10.1093/nar/gks842

**Published:** 2012-09-12

**Authors:** Christian B. Gerhold, Duane D. Winkler, Kristina Lakomek, Florian U. Seifert, Sebastian Fenn, Brigitte Kessler, Gregor Witte, Karolin Luger, Karl-Peter Hopfner

**Affiliations:** ^1^Department of Biochemistry, Gene Center of the Ludwig-Maximilians-University Munich, Feodor-Lynen-Str. 25, D-81377 Munich, Germany, ^2^Department of Biochemistry and Molecular Biology Howard Hughes Medical Institute and Colorado State University, Fort Collins, CO 80523, USA and ^3^Center for Integrated Protein Sciences (CIPSM), Gene Center of the Ludwig-Maximilians-University Munich, Feodor-Lynen-Str. 25, D-81377 Munich, Germany

## Abstract

Nuclear actin-related proteins (Arps) are subunits of several chromatin remodelers, but their molecular functions within these complexes are unclear. We report the crystal structure of the INO80 complex subunit Arp8 in its ATP-bound form. Human Arp8 has several insertions in the conserved actin fold that explain its inability to polymerize. Most remarkably, one insertion wraps over the active site cleft and appears to rigidify the domain architecture, while active site features shared with actin suggest an allosterically controlled ATPase activity. Quantitative binding studies with nucleosomes and histone complexes reveal that Arp8 and the Arp8–Arp4–actin-HSA sub-complex of INO80 strongly prefer nucleosomes and H3–H4 tetramers over H2A–H2B dimers, suggesting that Arp8 functions as a nucleosome recognition module. In contrast, Arp4 prefers free (H3–H4)_2_ over nucleosomes and may serve remodelers through binding to (dis)assembly intermediates in the remodeling reaction.

## INTRODUCTION

Chromatin remodeling and modifying complexes regulate or facilitate chromosomal processes such as transcription, replication and DNA repair by changing the position, spacing, histone composition or histone modification pattern of nucleosomes. A notable, poorly understood feature of several large multi-subunit chromatin modifying or remodeling complexes is the presence of nuclear actin-related proteins (Arps) (1) along with nuclear actin (2). Nuclear Arps associate in specific and unique combinations within the multi-subunit chromatin remodeling complexes INO80 (3), SWR1 (4), SWI/SNF (5), RSC (6) and the histone actetyl transferase NuA4 (7). The mechanistic role of nuclear Arps 4–9 in these complexes is not well understood (8).

INO80 contains actin along with Arp4, Arp5 and Arp8. Actin, Arp4 and Arp8 together with the helicase-SANT-associated (HSA) domain of the Ino80 ATPase form a stable sub-module of INO80 (9), while Arp5 together with INO80 subunits Ies2 and Ies6 associate with the AAA^+^ ATPase subunits Rvb1/Tip49 and Rvb2/Tip48 (10). The structure of Arp4 displays a conserved actin core fold and a high-affinity ATP-binding site, as well as several loop insertions or deletions at positions that are important for actin to form filaments (11). Consistently, Arp4 does not form multimers itself and together with Arp8 helps sequester and retain monomeric actin in the remodeler.

Arp4 is essential in yeast; Arp5 and 8 are indispensable for INO80 function since deletions of these genes mimic the *ino80*Δ phenotype (12). Deleting Arp8 in *Saccharomyces cerevisiae* results in an INO80 complex that also lacks Arp4 and actin, in addition to the losses of DNA binding and ATPase activities (12). *Arp8*Δ strains are defective in DNA repair and cell-cycle progression (13–15). Although INO80 in *S. cerevisiae* is recruited to DNA damage sites via Arp4 and Nhp10 in a H2A P-Ser129 dependent manner (*S. cerevisiae* does not have H2A.X) (16,17), mammalian INO80 seems to be targeted to γ-H2A.X foci exclusively by its Arp8 subunit, suggesting that the recognition of DNA damage by INO80 might differ across species (18). Arp8 also seems to fulfil additional functions independent of INO80 (19).

Recent results suggest that the INO80 complex at least in part acts in genome expression and maintenance through direct regulation of the genome-wide distribution of unacetylated H2A.Z, with Arp8 being involved in this process (20). Moreover, INO80 strongly binds to nucleosomes with extranucleosomal DNA and appears to function as a nucleosome spacing factor (21), but the exact contribution of individual INO80 subunits to nucleosome binding is not known. Interestingly, INO80 harbours a DNA-binding domain (DBINO) that coincides with the Arp8-Arp4-actin-binding HSA domain (9,22). Qualitative experiments indicate that Arp8 binds all four core histones, but prefers H3 and H4 over H2A and H2B in salt washes (12). However, it is unclear whether Arp8 and other Arps prefer free histones or nucleosomes, and how they interact with different histone complexes in a quantitative manner.

To provide a first structural framework for Arp8 we determined the crystal structure of *Homo sapiens* Arp8 (a truncated variant that lacks the first 33 N-terminal amino acids), and studied the solution structure of truncated and full-length human Arp8 (hArp8) by small angle X-ray scattering (SAXS). We found that hArp8, like Arp4, stably binds ATP. Long loop insertions embrace and conformationally lock the actin fold, which may account for the stable ATP-bound conformation and lack of polymerization capability of Arp8. In contrast to Arp4, we found that Arp8 has a low basal ATPase activity, suggesting that ATP-hydrolysis could contribute to Arp8 function. To dissect the role of Arp8 and Arp4 in targeting the INO80 complex to chromatin, we quantitatively analysed the binding of Arp8, Arp4 and the intact Arp8–Arp4–actin-HSA sub-complex I to the histone complexes H2A–H2B, (H3–H4)_2_, DNA and to nucleosomes. Our data show that Arp8 binds (H3–H4)_2_ with high affinity, and this property is to a large part responsible for its interaction with nucleosomes. In contrast, Arp4 shows a preference for free (H3–H4)_2_ over intact nucleosomes, and this property may facilitate recognition and/or association with partially remodeled nucleosomes. The binding of sub-complex I to nucleosomes can be dissected into contributions of Arps and the HSA/DBINO domain.

## MATERIALS AND METHODS

### Cloning, protein expression and purification

Human Arp8 full-length, yeast Arp8, Arp4 and INO80’s sub-complex I were expressed in HighFive^TM^ insect cells and human Arp8 (Δ1–33) in *Escherichia coli* BL21(DE3) cells. Proteins were purified using metal-affinity, anion exchange and size exclusion chromatography.

### *Homo sapiens* Arp8 (Δ1–33)

Human macrophage cDNA was used for amplification of the human Arp8 gene construct. Primers induced an NcoI and NotI restriction site as well as a sequence coding for an N-terminal 8xhis affinity tag and a Prescission Protease cleavage site into the gene fragment (hArp8; residues 34–624). The gene was subsequently cloned into the pET-28 vector (Novagen) and transformed into *E. coli* BL21(DE3) cells. Protein was expressed using standard methods at 18°C overnight. Cells were harvested by centrifugation at 2000*g* for 15 min and the resulting cell pellet stored at −20°C until required.

The cell pellet from 18 l expression culture was re-suspended in 100 ml of buffer A [20 mM Tris-HCl pH 8.0, 100 mM NaCl, 5 mM β-mercaptoethanol and 5% (v/v) glycerol] supplemented with protease inhibitors (Roche, Penzberg, Germany). Cells were lysed by sonication and cell debris was removed by centrifugation (40 000*g*, 45 min, 4°C). The supernatant was incubated for 1 h with Ni–NTA resin (Qiagen) at 10°C and subsequently purified using gravity flow with three wash steps of three column volumes buffer A containing 0, 20 and 35 mM imidazole. Bound protein was eluted with buffer A containing 250 mM imidazole. The eluate was applied to a Q sepharose column (GE Healthcare) and the flow-through containing Arp8 was supplemented with PreScission Protease (GE Healthcare) to cleave off the N-terminal affinity tag and dialysed against buffer A overnight at 4°C. The protein solution was centrifuged (40 000*g*, 20 min, 4°C) and the supernatant was incubated with GSH sepharose (GE Healthcare) to remove GST-fused PreScission protease.

The flow-through was again applied to a Ni–NTA resin and the obtained new flow-through was further purified via size exclusion chromatography with a Superdex-200 (GE Healthcare). The protein was concentrated up to 14 mg/ml, flash-frozen in liquid nitrogen and stored at −80°C until required.

### Full-length *Homo sapiens* Arp8

The gene was amplified from human macrophage cDNA with primers containing SalI and NotI restriction enzymes and coding for an N-terminal hexahistidine tag and cloned into the pFBDM vector (Redbiotech, Schlieren/Switzerland) and protein expression was achieved according to a published protocol (23). The purification protocol was similar to that for the N-terminally truncated hArp8 construct but without PreScission protease cleavage and subsequent GSH- and second Ni–NTA purification.

### *Saccharomyces cerevisiae* Arp4 and Arp8

Cloning, expression and purification were performed as described previously (11).

### *Saccharomyces cerevisiae* sub-complex I

*Saccharomyces cerevisiae* Arp4, Arp8, actin and Ino80 (462–685) were cloned into the pFBDM vector. A sequence coding for an N-terminal hexahistidine tag was added to Ino80 (462–685). Two plasmids were merged to a single pFBDM vector carrying all four genes of interest and protein expression in HighFive™ cells (Invitrogen) was accomplished according to a published protocol (23). The protein sub-complex I was purified similarly to full-length human Arp8 with the exception that a linear gradient with buffer B [20 mM Tris-HCl pH 8.0, 1 M NaCl, 5 mM β-mercaptoethanol and 5 % (v/v) glycerol] was applied to elute the sub-complex from the Q sepharose column (GE Healthcare).

### Crystallization, data processing, structure determination and refinement

#### Crystallization of human Arp8 (33–624)

Crystals of human Arp8 (Δ1–33) were grown at 16°C in 3.9 M NaCl, 0.5% (v/v) methanol, 50 mM (2-(N-morpholino)ethanesulfonic acid) MES pH 6.1, mixed 1:1 with 4.5 mg/ml protein solution using hanging drop vapour diffusion. Seeding with these small but regular human Arp8 crystals yielded rod shaped crystals of up to 500-µm length after 4–5 weeks at 10°C. Crystals were cryoprotected for data collection in a buffer containing 50 mM MES pH 6.1, 3.5 M NaCl and 25% (v/v) glycerol.

#### Data collection and processing, structure determination and refinement

Diffraction data to a resolution of 2.6 Å were collected on a single crystal at 100 K and a wavelength of 1.0 Å at beamline X06SA (Swiss Light Source, Villingen, Switzerland). Data were processed and scaled with XDS and XSCALE (24) in space group 20 (C222_1_). The molecular replacement model was derived from the structure of yeast Arp4 (pdb: 3QB0) with all non-identical residues cut at the β-carbon atom using CHAINSAW (25). Molecular replacement was then carried out with PHASER (26) with one molecule of human Arp8 per asymmetric unit. An initial model of high quality was obtained using cycles of automated model building with ARP/wARP (27) and BUCCANEER (28) in the CCP4 Suite (29) and completed by manual building using COOT (30). Refinement with PHENIX (31) finally resulted in a 2.6 Å structure with good stereochemistry and reasonable R-factors of *R*_work_/*R*_free _16.0/20.4% (Table 1).

**Table 1.** Data collection and refinement statistics

Data collection	
Space group	C222_1_
Cell dimensions	
*a*, *b*, *c* (Å)	80.88 151.26 173.35
α, β, γ (°)	90, 90, 90
Resolution (Å)	86.68–2.59 (2.75–2.59)^a^
*R*_obs_ (%)	7.9 (67.0)
*I* / σ(*I*)	14.16 (2.17)
Completeness (%)	99.2 (98.8)
Redundancy	3.6 (3.5)
Refinement	
Resolution (Å)	44.90–2.60 (2.68–2.60)
No. reflections	32 837
*R*_work_ / *R*_free_	16.34 / 20.95
No. atoms	
Protein	4051
Ligands/ions	93
Water	146
*B* factors (Å^2^)	
Protein	50.04
Ligands/ions	55.38
Water	44.84
R.m.s. deviations	
Bond lengths (Å)	0.008
Bond angles (°) Ramachandran plot (%)	1.185
Favored	97.4
Allowed	2.6
Outliers	0.0
PDB Accession code	4FO0

^a^Values in parentheses are for highest-resolution shell.

The simulated annealing 2*F*_o_*F*_c_ omit map for a bound ATP molecule (Supplementary Figure S1) was calculated using CNS (32,33). Coordinates have been deposited in the Protein Data Bank (accession code 4FO0). Images for publication were generated using PyMol (34) and Chimera (35).

### ATPase assay

Reactions were performed in 50 mM Tris–HCl pH 7.9, 100 mM NaCl, 2 mM MgCl_2_, 2 mM dithiothreitol (DTT) and 100 µM ATP including 5 nM radioactively labelled [γ-^32^P]ATP (Hartmann Analytic, Braunschweig). Increasing concentrations of protein (yeast Arp4, yeast Arp8, yeast sub-complex I, human Arp8) were incubated in presence or absence of nucleosomes or constituents thereof at 30°C for 30 min or 4 h. Free phosphate was separated by thin-layer chromatography on TLC PEI Cellulose F (Merck, Darmstadt) with 1 M formic acid containing 0.5 M LiCl, then incubated on storage phosphor screens (GE Healthcare, Heidelberg) for at least 3 h. Phosphorescence was scanned on a STORM 860 Scanner (Molecular Dynamics, Sunnyvale, CA, USA) and images were analysed using ImageJ.

### Small angle X-ray scattering

Human Arp8 protein samples for SAXS measurements were purified as stated above. Flow-through of the concentration step was used as buffer reference for the measurements.

SAXS data were collected at beamline X33, EMBL/DESY (Hamburg, Germany) and ID14-3, ESRF (Grenoble, France) at a cell temperature of 20°C.

The molecular weight of human Arp8 samples in solution was estimated according to Porod-volume analysis (36) (Supplementary Table S1). Human Arp8 was measured at a protein concentration of 6.15 mg/ml (full-length) or 1.5 mg/ml (truncated construct). Data were processed with the ATSAS package (37) and Guinier analysis yielded a radius of gyration *R*_g_ = 3.1 nm and showed no signs of aggregation. The Kratky plot shows a bell-shaped curve indicating that hArp8 is folded in solution (36). A set of 16 independent *ab initio* structures was calculated using GASBOR without any symmetry information given. One representative GASBOR model was chosen for representation (Supplementary Figure S7A) as all *ab initio* models were highly similar [nominal spatial discrepancy values of NSD ∼1.0 e.g. (38)] and the bead model was transformed to an electron density using the SITUS package (39). The theoretical scattering data of the atomic model were calculated with CRYSOL (40) and compared to the experimental scattering data (Figure 4).

### Nucleosome affinity assays

The microplates were prepared by sequential washing with 1 M HCl, 1% Hellmanex and Sigmacote. Each wash step was incubated for 30 min and followed by rinsing with distilled water. The plates were then air-dried under an exhaust hood for 8 h to overnight. The individual binding experiments were derived from titration of a highly concentrated stock of the recombinant Arp constructs and complexes (assay concentrations ranging from 1 to 5000 nM) into the fluorescently labelled histone complexes or nucleosomes. The H2A–H2B and H3–H4 complexes were labelled through Alexa 488 maleimide conjugation to H2B T112C or H4 E63C, respectively. The 30-bp ‘601’ sequence linker DNA was first modified with a 5′ C6 amine then conjugated with a succinimidyl ester derivative of the Atto 647N dye (Sigma-Aldrich, Munich). The fluorescently labelled DNA was then agarose gel purified to remove excess free dye. The two-step labelling procedure has previously been described elsewhere (41).

The labelled histones, nucleosomes, etc. were kept at a constant concentration between 0.5 and 1 nM with a final volume of 40 µl. The reaction conditions were maintained at: 20 mM Tris pH 7.5, 150 mM KCl, 5% (v/v) glycerol, 1 mM TCEP, 0.01% (w/v) CHAPS and 0.01% (w/v) octylglucoside. The titrations were then allowed to equilibrate at room temperature for 20–30 min (in the dark) and then scanned in-plate using a Typhoon 8600 variable mode fluorimager. Specific binding events were considered as a function of the fluorescence change (whether positive or negative) across the titration series. Interactions with affinities >5000 nM are here considered non-specific. The fluorescence change upon binding must be >10% of the total fluorescence signal to be considered for further evaluation. The actual fluorescence signal change was quantified using the program ImageQuant TL. Data analysis and non-linear fitting of the data was done with Graphpad Prism. All experiments were performed in replicative quadruplicates. For a more detailed explanation of the equations and reaction schemes refer to our previous works (42,43).

## RESULTS AND DISCUSSION

### Structural alterations of Arp8 compared with actin lead to different properties

**Figure 1.**
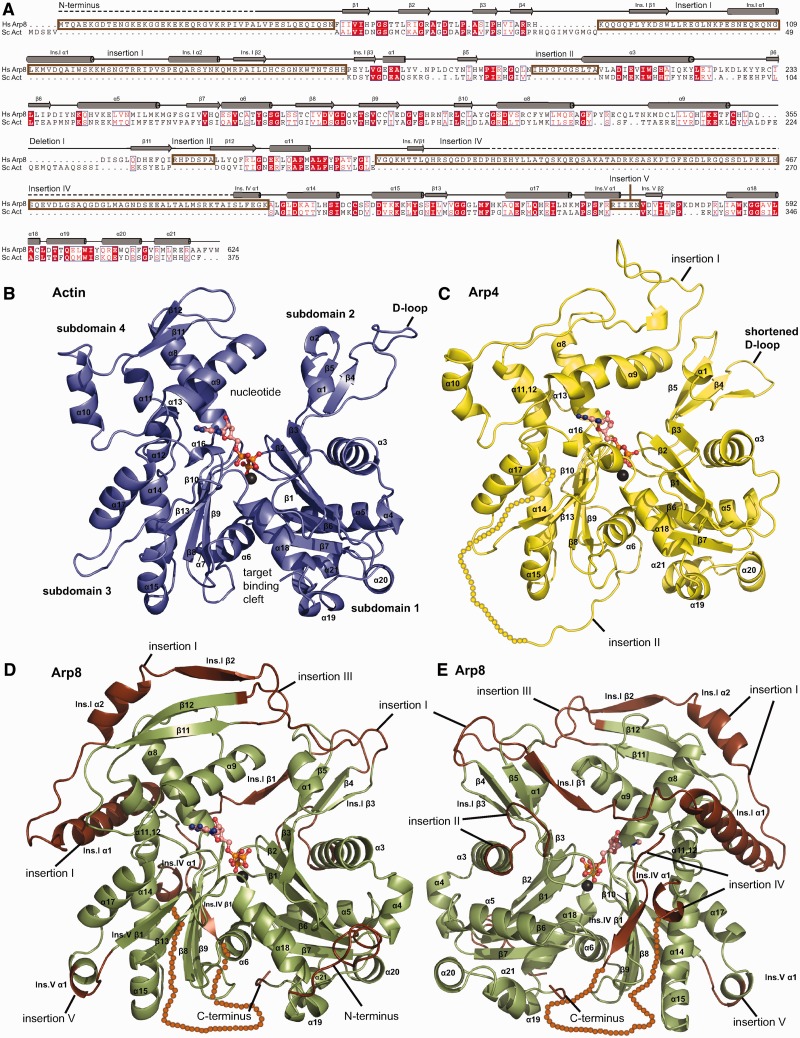
Alignment and structure comparison of *H. sapiens* Arp8 with actin. (**A**) Structure-based amino acid sequence alignment of human Arp8 with actin reveals five sequence insertions and one deletion. (**B**) Classical view of ATP-actin (blue) with the nucleotide-binding cleft at the pointed and the target-binding cleft at the barbed end of the molecule. Sub-domains 1 and 2 comprise the outer or small domain while sub-domains 3 and 4 are annotated inner or large domain. (**C**) Structure of ATP-bound *H. sapiens* Arp8 (Δ1–33) (green) in the classical actin view with insertions depicted in brown. The basal actin fold of Arp8 can be recognized and is complemented by five insertions. Insertions I emanates from the DNase I-binding loop and covers actin’s pointed end like a lid. Insertion III aids in closing the nucleotide-binding cleft and insertion V adds a small α-helix to sub-domain 3. (**D** and **E**) The backside of Arp8 shows that insertion II adds a loop and one turn to the α-helix that separates sub-domains 1 and 2. Insertion IV emanates from the region of actin’s hydrophobic plug but could not be allocated to electron density.

We crystallized a N-terminally truncated *H. sapiens* Arp8 construct (Δ1–33) in space group C222_1_ and determined its crystal structure to 2.6 Å resolution using molecular replacement phasing with the structure of Arp4 (3QB0) as a search model (Table 1 and Figure 1C). The crystals contained one Arp8 molecule per asymmetric unit. Except for 91 amino acids (R411–I501) in a loop insertion, we obtained interpretable electron density for the entire protein. Automatic as well as manual model building and refinement resulted in a model with good stereochemistry and *R*-factors. Additional density in the nucleotide-binding cleft could be interpreted as a bound ATP molecule with one coordinated metal ion (Supplementary Figure S1).

Despite the relatively low sequence homology to actin, human Arp8 displays the typical actin fold with a central nucleotide-binding cleft between sub-domains 1 and 2 on one side and sub-domains 3 and 4 on the other side (Figure 1B). However, Arp8 has several striking as well as subtle structural differences when compared to actin (Figure 1D, E and Supplementary Figure S2) (44), and these insertions are mostly located at the four ‘hot spots’ for Arp insertions (45). The most notable feature of Arp8 is a long insertion I (K80–H162), which emanates from the ‘DNaseI binding loop’ (D-loop) on sub-domain 2, binds across both halves of the actin fold to wrap around sub-domain 4 and reaches back to the D-loop. Insertions II and III interact with insertion I and together, these three insertions cover the ‘pointed end’ of Arp8. We find several surface exposed hydrophobic residues as well as bound glycerol from the crystallization solution, suggesting these regions are involved in protein–protein interactions. Several secondary structure elements in insertion I make intimate contact with sub-domain 4, e.g. Ins.I α1 that forms part of the helix bundle of sub-domain 4 and Ins.I β1 and 2 that attach as additional strands to the β-sheets of sub-domains 2 and 4. All in all, insertions I–III appear to lock the two principal domains of actin (sub-domains 1–2 and sub-domains 3–4) into a particular conformation and stabilize the active site cleft. This is in stark contrast to actin where both principal domains have much less direct interaction, and conformational changes between these domains distinguish ADP from ATP-bound forms in G-actin and F-actin.

The second major insertion (IV) (V401–K507) emanates from what comprises actin’s hydrophobic plug and is therefore situated on the side of actin that participates in filament formation. This insertion did not give rise to interpretable electron density except for a few residues suggesting that it is highly flexible and could mediate protein–protein interactions. The subsequent insertion V (R565–N569) is a relatively small insertion that introduces a short α-helix into the actin structure at the outer end of the barbed end of sub-domain 3 (Figure 1D and E).

In summary, Arp8 shows the flattened disk like shape of actin with concave and convex sides. While the concave side only carries small insertions, the Arp8 characteristic insertions cover the convex (filament facing side of actin) and pointed side of the molecule and explain why Arp8 itself does not form filaments. It is likely that these insertions in part evolved to mediate contacts with other subunits of the INO80 complex, suggesting that Arp8 uses the same filament facing side as actin for protein–protein contacts (Supplementary Figure S2).

### ATP is tightly bound to human Arp8

**Figure 2.**
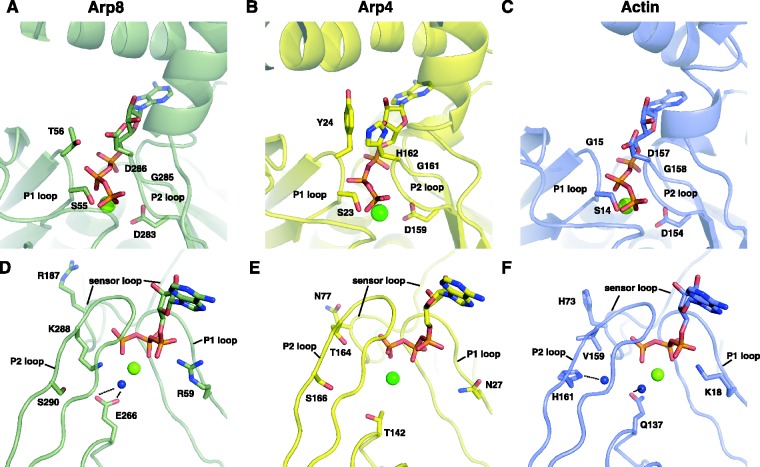
Close up of human Arp8’s active site. (**A** and **D**) Human Arp8 coordinates ATP similar to actin (see **C**) and E266^Arp8^ could activate the ATPase akin to Q137^act^. This would require an additional factor that binds to Arp8’s target-binding cleft. The H73^act^ sensor is replaced by R187^Arp8^, which could in principle trigger a similar conformational change. (**B** and **E**) ATP in yeast Arp4 is more shielded compared to actin or Arp8. Also, T142^Arp4^ is probably too far apart from the γ-phosphate for a putative ATPase activation. Hence, ATP seems to play an exclusively structural role in Arp4. **(C** and **F**) ATP in actin (pdb: 1YAG) is bound by residues in the P1 and P2 loops and activation of the ATPase is triggered by Q137^act^, which comes into closer proximity to the γ-phosphate upon filament formation. After hydrolysis and release of the γ-phosphate S14 flips over to coordinate the β-phosphate making room for H73 of the sensor loop to occupy the now available space. This conformational change propagates via sub-domain 2 to the D-loop.

Electron density in the nucleotide-binding cleft of actin could clearly be assigned to ATP as well as a metal ion (likely Mg^2+^) that coordinates the β- and γ-phosphates of the nucleotide (Supplementary Figure S1 and Figure 2A). Since ATP was not added prior to crystallization, it was retained from the cells throughout purification and crystal growth. Thus human Arp8, at least apart from INO80 or other binding partners, lacks significant ATP hydrolysis activity albeit it very strongly binds ATP.

The nucleotide-binding site in Arp4 was significantly altered compared to actin’s active site, proposing a structural function for ATP in Arp4 (Figure 2B) (11). On the contrary, the nucleotide-binding site of Arp8 exhibits only minor changes to the residues involved in the catalytic cycle of actin’s ATPase (Figure 2C). G15^act ^of the P1 loop is replaced by T56^Arp8^, which could in principle aid S55^Arp8^ in complexing the β-phosphate, once hydrolysis and *P*_i_ release is completed. The main residues involved in metal ion coordination and nucleotide binding of the P2 loop remain the same in Arp8 compared to actin as D154^act^, G156^act^ and D157^act^ structurally align with D283^Arp8^, G285^Arp8^ and D286^Arp8^. Filament-activated Q137^act^ plays a critical role in ATP hydrolysis (46) (Figure 2D–F), since Q137^act^ is in closer proximity to the γ-phosphate in polymerized actin (Figure 2F) (47,48), and stimulates the ATPase >40 000-fold (49). In Arp8 this residue is altered to E266^Arp8^ (Figure 2D), which could fulfil similar tasks, if activation can occur. Even though the catalytically active H161^act ^is substituted by S290^Arp8^, a neighbouring K288^Arp8^ might play a role in the putative ATP hydrolysis in Arp8.

In actin, upon ATP hydrolysis and *P*_i_ release, S14^act^ rotates to contact the β-phosphate of the remaining ADP, allowing H73^act^ in the sensor loop to penetrate into the space that was previously occupied by the γ-phosphate (50). This movement influences the width of the nucleotide-binding cleft as well as the conformation of the D-loop, thus weakening the interaction with the adjacent intra-strand actin in the filament (51).

Histidine H73^act^ of the sensor loop is substituted by R187^Arp8^ in Arp8. It is conceivable that this arginine takes over the sensor part in Arp8 and mediates a similar conformational change along sub-domain 2. However, the acceptor of this movement is insertion I instead of the D-loop and the putative repositioning of residues would then spread along the lid that covers Arp8’s pointed end. Insertion II is also directly connected to the hArp8 sensor loop and might be an alternative or additional acceptor of conformational rearrangements.

In summary, the intact nucleotide-binding site suggests that Arp8, like actin, is an extremely weak ATPase in a monomeric state. However, a binding partner located at the highly conserved target-binding cleft (Supplementary Figure S4) could induce the subtle changes necessary to stimulate the ATPase that could be part of Arp8`s function.

### Arp8 shows low basal ATPase activity

**Figure 3.**
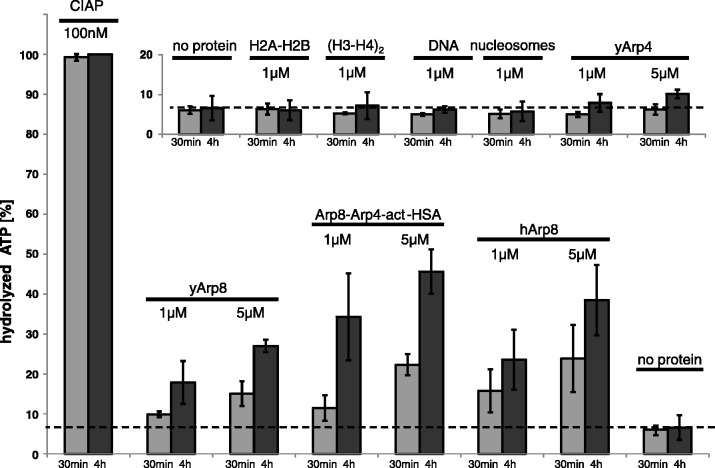
ATPase activity of actin-related proteins 4 and 8. Low basal ATPase activity was found for yeast and human Arp8 but not for yeast Arp4. The Arp8–Arp4–actin-HSA sub-complex I of INO80 has a slightly higher activity than Arp8 but does not show any sign of efficient stimulation of the ATPase of either Arp8 or actin within this sub-complex. Arp4, H2A–H2B dimers, (H3–H4)_2_ tetramers, DNA and nucleosomes have no measurable ATPase activity in comparison with the control reaction without proteins. No significant stimulation of the ATPase activity of Arp8 or sub-complex I was triggered by canonical nucleosomes and its constituents (Supplementary Figure S5).

We tested the structure-based hypothesis that Arp8 harbours weak ATPase activity by a ^32^P ATP hydrolysis assay. We found that yeast and human Arp8 possess a low basal ATPase activity, while Arp4 did not hydrolyse ATP above background (Figure 3 and Supplementary Figure S5). This argues that ATP in Arp4 has a more structural role, while ATP-hydrolysis could contribute to Arp8 function in INO80. The INO80 sub-complex I (Arp8–Arp4–actin-HSA) also shows a low basal ATPase activity, most likely due to Arp8 and actin. We then tested the binding partners H2A–H2B, (H3–H4)_2_, 146-bp DNA and nucleosomes (Supplementary Figure S5) for a potential stimulation or inhibition of the ATPase activity of Arp8 or sub-complex I. We did not find a significant stimulation or inhibition, arguing that Arp8’s ATPase is not allosterically regulated by the tested components (Supplementary Figure S5). However, we do no want to rule out that Arp8’s ATPase activity is regulated by INO80 subunits or histone variants, or alternatively post-translational modifications of the Ino80 subunits (52,53) or histones.

### Arp8 is a monomer in solution

A conserved and notable feature of Arp8 is the N-terminal extension. It is rather short in human Arp8 compared to yeast Arp8, but still comprises 46 mostly charged residues that emerge from the ‘barbed’ end of the molecule. Interestingly, we found that in the crystal lattice, two hArp8 molecules form a 2-fold symmetric dimer, where the root of the N-terminal extension of one hArp8 binds into a hydrophobic groove of the opposing hArp8 and vice versa (Supplementary Figure S6). These symmetrical crystal contacts and the partially hydrophobic interactions may indicate that Arp8 can form transient dimers, perhaps concomitantly recognizing two sides on the nucleosome.

**Figure 4.**
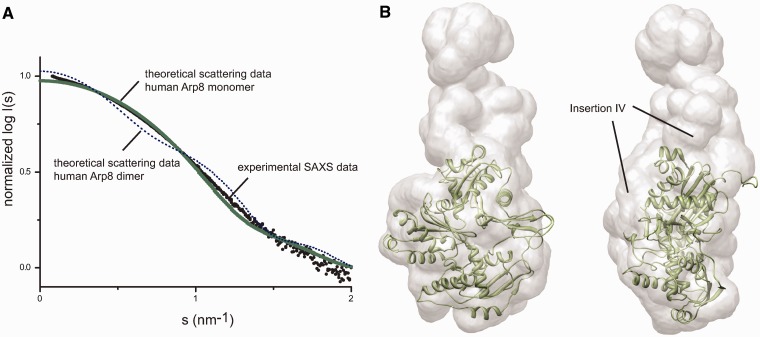
Solution structure of human Arp8. (**A**) Theoretical SAXS scattering curves calculated with CRYSOL for monomeric and dimeric human Arp8 show that Arp8 is a monomer in solution. (**B**) X-ray structure of human Arp8 (Δ1–33) docked into the *ab initio* SAXS model of full-length hArp8. The solution structure of human Arp8 provides extra density for insertion IV.

Therefore, we analyzed the solution structure of hArp8 by SAXS and SLS (static light scattering) to reveal possible Arp8 multimers. Consistent with our previous SAXS analysis of *S. cerevisiae* Arp8 (11), we found hArp8 to be monomeric in solution (Figure 4). The X-ray structure could be nicely docked into the SAXS dummy residue model, which shows the characteristic concave and convex sides of the flattened actin fold. Solution structures of full-length hArp8 and the crystallized construct differ only marginally (Supplementary Figure S7). Unaccounted regions of the dummy residue model could be allocated to the large loop insertion IV and N-terminal extension that are not part of the crystal structure model. The molecular weights calculated from the Porod volume are 78 kDa for full-length Arp8 and 70 kDa for the crystallized construct, consistent with a monomer (human Arp8 chain mass = 70.5 kDa). In addition, the theoretical scattering curve of monomeric hArp8 calculated with CRYSOL fits better to the measured curve than the theoretical scattering curve of a potential hArp8 dimer (Figure 4A). SLS also confirms Arp8, Arp4 and the sub-complex I to be monomeric entities in solution (Supplementary Figure S8). Thus, Arp8 alone is monomeric in solution, although it may have the potential for dimer formation (see below).

### Arp8, Arp4 and the INO80 sub-complex I bind (H3–H4)_2_ and nucleosomes with high affinity

**Figure 5.**
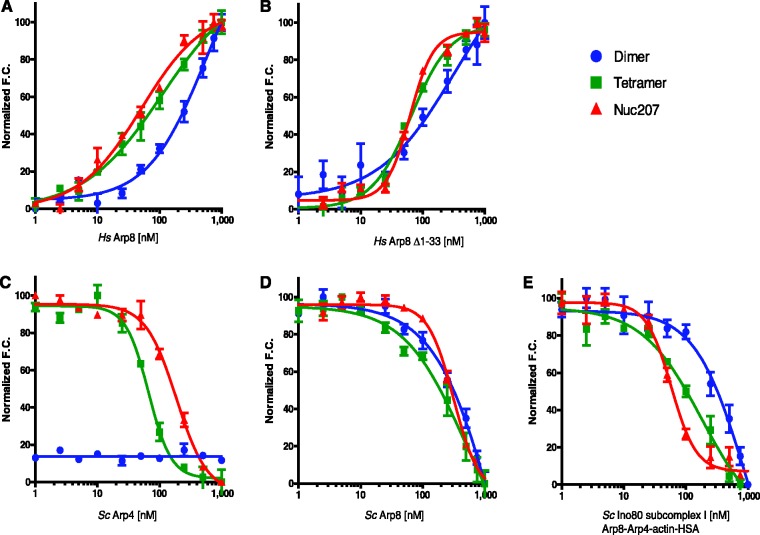
Contribution of Arps to histone and nucleosome binding. Fluorescence based affinity measurements of *H. sapiens* Arp8 full-length (**A**), human Arp8 (Δ1–33) (**B**), *S. cerevisiae* Arp8 (**C**), yeast Arp4 (**D**), *S. cerevisiae* INO80 sub-complex I (Arp8–Arp4–actin-HSA) (**E**) to H2A–H2B dimers (blue data points), (H3–H4)_2 _(green) and nucleosomes (red). Binding affinities and Hill coefficients are determined by titration of Arps or complex into fluorescently labelled histones or nucleosomes, and monitoring of fluorescence change over the titration series. Change of fluorescence (increase or decrease) upon substrate binding depends on alterations to the microenvironment of the attached fluorophore upon specific binding events.

To investigate the role of the Arp subunits in mediating the interaction of INO80 with chromatin, we used an established quantitative method (54) to measure their interactions with histone complexes H2A–H2B and (H3–H4)_2_, DNA and with nucleosomes. We assayed full-length and N-terminally truncated human Arp8 as well as yeast Arp8, Arp4 and the Arp8–Arp4–actin-HSA sub-complex I (Figure 5 and Table 2).

**Table 2.** The calculated dissociation constants (*K*_d_^app^), Hill coefficients (*n*_H_), and overall non-linear fit of the data (*R*^2^) for *H. sapiens* (*Hs*) Arp8 (Δ1–33), *Hs*Arp8, *S. cerevisiae* (*Sc*) Arp4, *Sc*Arp8 and *Sc* INO80 sub-complex I

	*K*_d_^app ^(×10^−9^ M)	Hill coefficient	Overall fit (*R*^2^)
*Hs* Arp8 (Δ1–33)		
30 bp DNA	6329 *± *2727	2.4 *± *1.1	0.97
H2A–H2B	367 *± *131	0.76 *± *0.3	0.85
H3–H4	65.1 *± *6.4	1.37 *± *0.2	0.97
207-bp nucleosome	62.6 *± *16	2.11 *± *0.4	0.91
*Hs* Arp8			
30-bp DNA	6938 *± *3448	2.6 *± *0.6	0.95
H2AH2B	555 *± *158	0.91 *± *0.2	0.92
H3–H4	110 *± *40.4	1.11 *± *0.3	0.96
207-bp nucleosome	51.0 *± *9.6	1.31 *± *0.4	0.96
*Sc* Arp4			
30 bp DNA	NB	–	–
H2A–H2B	NB	–	–
H3–H4	74.3 *± *10	2.41 *± *0.6	0.89
207-bp nucleosome	204 *± *67	2.06 *± *0.7	0.85
*Sc* Arp8			
30-bp DNA	1919 *± *182	1.7 *± *0.2	0.99
H2A–H2B	1951 *± *796	0.85 *± *0.2	0.95
H3–H4	485 *± *196	0.87 *± *0.2	0.92
207-bp nucleosome	314 *± *35	2.13 *± *0.3	0.98
*Sc* Arp8–Arp4–actin-HSA			
30-bp DNA	366 *± *23	2.1 *± *0.3	0.99
H2A–H2B	853 *± *358	1.91 *± *0.8	0.81
H3–H4	182 *± *66.4	1.01 *± *0.2	0.95
207-bp nucleosome	63.6 *± *6.2	3.13 *± *0.7	0.94

NB: no binding determined.

All Arp8 variants bind (H3–H4)_2_ and nucleosomes with comparable affinity, while the interaction with H2A–H2B is ∼4- to 6-fold weaker. The values obtained for truncated human Arp8 are comparable to those for full-length human Arp8. *S. cerevisiae* Arp8 follows similar trends in binding nucleosomes and histone complexes as *H. sapiens* Arp8, but binds with overall lower affinity compared to the human protein. This may imply differences in chromatin targeting (16–18). Alternatively, this may be observed as the *Xenopus laevis* histones used here are more compatible with human Arp8 than with yeast Arp8, or because there might be less active protein in this particular preparation. All binding curves for histones exhibit Hill coefficients near 1. In contrast, each version of Arp8 binds nucleosomes with a Hill coefficient >1 (Table 2). This suggests cooperative binding of more than one Arp8 molecule per nucleosome, and implies that binding of the first Arp8 molecule facilitates subsequent binding events. These data lend credence to the idea that Arp8 may form multimers upon nucleosome binding.

In contrast to Arp8, Arp4 exhibits a clear preference for (H3–H4)_2_ over nucleosomes, and binds both substrates with a Hill coefficient that indicates cooperativity. The interaction of Arp4 with H2A–H2B is too weak to be determined reliably. Together, these data uncover unique properties of the two Arps and suggest that Arp4 interacts with regions of the H3–H4 tetramer that are at least partially masked in the context of a nucleosome.

Finally, we probed the histone and nucleosome-binding properties of the INO80 sub-complex Arp8–Arp4–actin-HSA. This complex exhibits a 3-fold higher affinity for nucleosomes compared to (H3–H4)_2_. It binds nucleosomes with a Hill coefficient of 3, while its interaction with (H3–H4)_2_ exhibits no signs of cooperativity. Again, the affinity for H2A–H2B dimer is very low. We conclude that the contribution of Arp8 to the nucleosome-binding ability of INO80 is significant. Arp8 may require distinct nucleotide states for optimal binding. Because Arp4 tightly binds to the histone (H3–H4)_2_ complex with a *K*_d _of 70 nM, which is higher than the affinity of the sub-complex for this substrate (182 nM), potent (H3–H4)_2_ binding by Arp4 appears to be compromised in the sub-complex. Both human and yeast Arp8 bind to DNA with low affinity in the micromolar range, while no interaction with DNA was observed for Arp4. The sub-complex I on the other hand has substantial DNA-binding activity (366 nM), which most likely is accounted for by the HSA/DBINO domain with a potential minor contribution of Arp8 (Table 2 and Supplementary Figure S9). As it has been shown that INO80 prefers nucleosomes with extranucleosomal DNA over nucleosomes without linker DNA (21), the affinity of the sub-complex I to DNA is likely to contribute to chromatin binding of INO80.

In summary, the structure of Arp8 reveals similarities but also important differences to actin. Most notably, we found an active site that stably binds ATP but, according to similarities with actin, may have an allosterically regulated ATPase activity. Loop insertions rigidify the actin fold and may transmit any conformational changes due to nucleotide status to interaction partners. The binding assays of Arp8, Arp4 and sub-complex I elucidate quantitative differences in their interaction with nucleosomes and histone complexes. The data suggest that Arp8 targets intact nucleosomes in the context of INO80, while Arp4 may act as chaperone in the interaction with free or partially exposed (H3–H4)_2_. This property may come into play in the context of either SWR1 or NuA4, where Arp4 and actin are also found.

## ACCESSION NUMBERS

The PDB ID Code: 4FO0.

## SUPPLEMENTARY DATA

Supplementary Data are available at NAR Online: Supplementary Table 1, Supplementary Figures 1–9 and Supplementary References [55,56].

## FUNDING

The Deutsche Forschungsgemeinschaft (DFG) [SFB TR5 to K.-P.H. and GRK1721 to K.-P.H. and G.W.]; the National Institutes of Health [GM088409 to K.L.]; National Research Service Award [F32GM096531 to D.D.W.]; the Howard Hughes Medical Institute (to K.L.); the Center for Integrated Protein Science (to K.-P.H.). Funding for open access charge: DFG [GRK1721].

*Conflict of interest statement*. None declared.

## Supplementary Material

Supplementary Data
