# Supplementary material for: Structure of Actin-related protein 8 and its contribution to nucleosome binding
Source: Nucleic Acids Res. 2012 Sep 12;40(21):11036–46. doi: 10.1093/nar/gks842 (PMC3510490; doi:10.1093/nar/gks842)
Supplement: Supplementary Data [file supp_40_21_11036__index.html]

Structure of Actin-related protein 8 and its contribution to nucleosome binding — Structure of Actin-related protein 8 and its contribution to nucleosome binding — Supplementary Data 

# Structure of Actin-related protein 8 and its contribution to nucleosome binding

## Supplementary Data

files

**Files in this Data Supplement:**

- Supplementary Data - pdf file
