# Supplementary material for: Structure of Actin-related protein 8 and its contribution to nucleosome binding
Source: Nucleic Acids Res. 2012 Sep 12;40(21):11036–46. doi: 10.1093/nar/gks842 (PMC3510490; doi:10.1093/nar/gks842)
Supplement: Supplementary Data [file supp_gks842_nar-00989-m-2012-File007.pdf]

Supplemental information

ATP is bound to human Arp8

Crystallized human Arp8 harbored ATP in its binding site, which was purified from the expression host and was not hydrolyzed during purification and crystal growth.

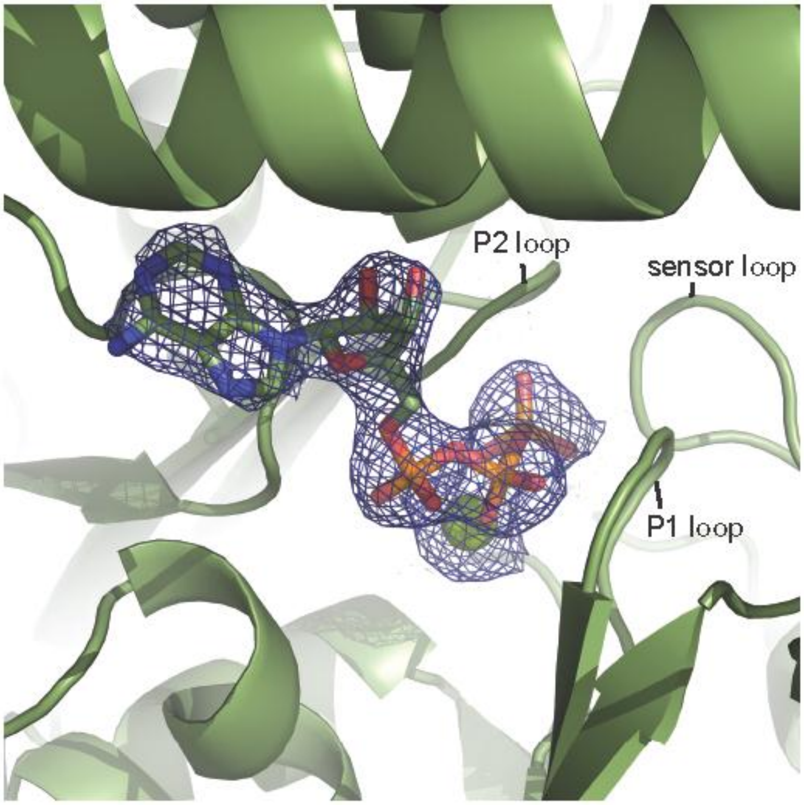

Figure S1: Electron density for ATP bound to human Arp8.
The blue mesh corresponds to the electron density of a simulated annealing $2F_oF_c$ omit map contoured at 5.0 σ. The bound ATP molecule is shown in stick representation and the metal ion as sphere (light green). Surrounding regions of Arp8 harboring the nucleotide coordinating residues are represented in cartoon mode (green).

Several insertions change the surface of Arp8 compared to actin

The surface characteristics of Arp8 impair polymerization despite its basal actin fold. Five insertions and one charged knob markedly alter the actin core of Arp8. Interestingly, the barbed end comprises only one minor change due to the small insertion V. Also, the side of the actin fold that faces outward of the actin-filament structure (A) is only slightly modified. A charged knob (K577,D578, M579) and the elongated N-terminus are the only additional features of Arp8 to the actin fold on this side. The pointed end as well as the back side of the classical view of the actin fold (C) are both significantly changed. Insertion I spans over the whole width of the protein on this side to fold back onto the pointed end and insertion IV emanates from the so called hydrophobic plug. Moreover, insertion II is also located on this side of the actin fold. These modifications play a major role in the incapability of Arp8 to form filaments.

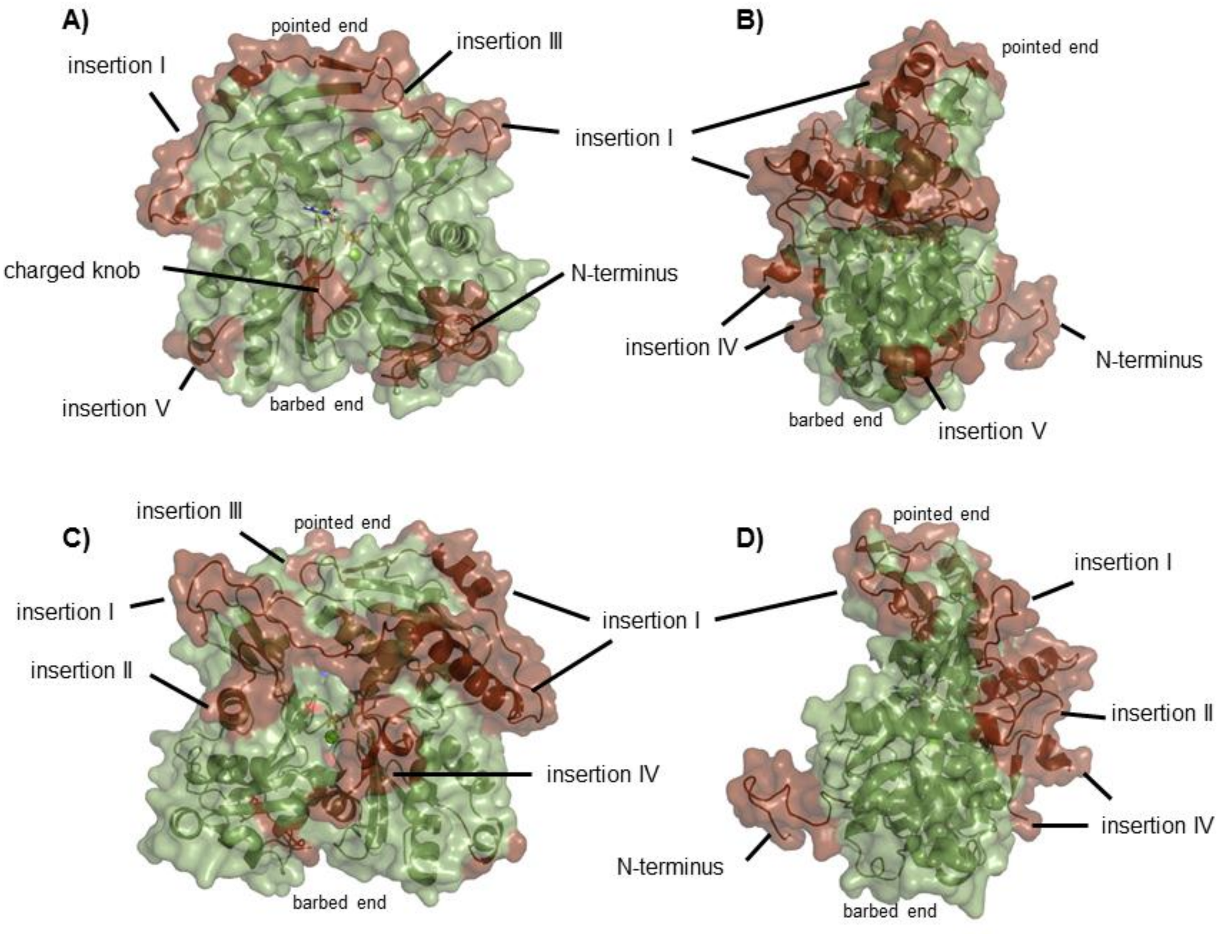

Supplementary Figure S2: Surface representations of human Arp8 (Δ1-33).
Surface presentations highlight the insertions of Arp8 (brown) compared to the basal actin fold (green). (A) The classical view on the actin fold depicts the closure of the pointed end of hArp8, but otherwise only minor changes to the surface of the actin fold. (B) View onto the inner domain of the actin fold. Here, insertion I markedly alters the surface at the pointed end side of the inner domain. (C) Rear view of the actin fold. Insertion I reaches over this “filament-facing” side of actin and insertion IV, which mostly lacks in the density emanates from the hydrophobic plug of the basal actin fold, (D) The view onto the outer domain of the actin fold shows only minor changes compared to the actin core.

Sequence conservation of Arp8

Arp8 is conserved from yeast to man with the exception of algae, Apicomplexa and the two Metazoan phyla *Caenorhabditis elegans* and *C. intestinalis*.

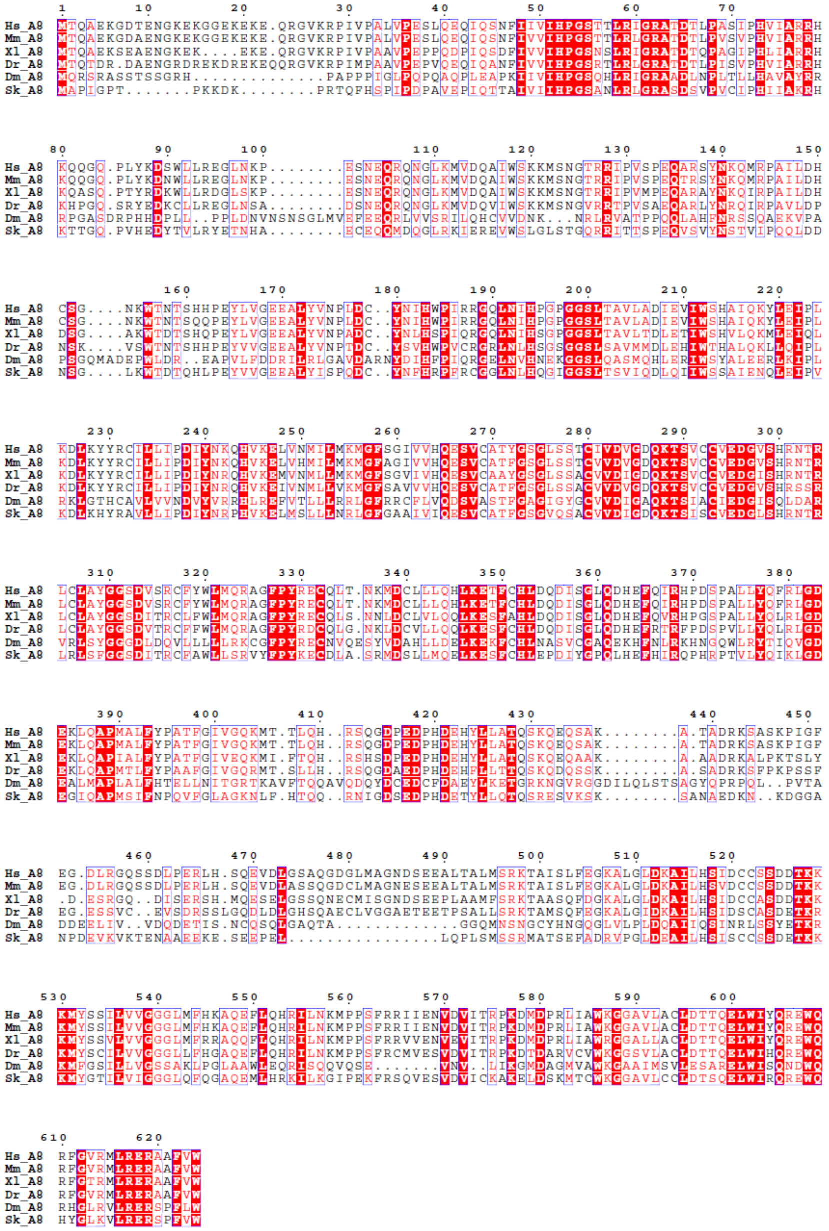

Supplementary Figure S3: Multiple sequence alignment of Arp8.
Amino acid sequences corresponding to homologues of human Arp8 were obtained from the NCBI protein database (http://ncbi.nlm.nih.gov). Abbreviations are defined as follows: Hs, *homo sapiens*; Mm, *mus musculus*; Xl, *Xenopus laevis*; Dr, *Danio rerio*; Dm, *Drosophila melanogaster*; Sk, *Saccoglossus kowalevskii*. The multiple sequence alignment was generated using ClustalW (55) (http://www.ebi.ac.uk/Tools/msa/clustalw2/) and formatted for presentation with ESPript (http://espript.ibcp.fr/ESPript/ESPript). The amino acid sequence numbers correspond to the human protein.

Amino acid conservation of Arp8 in the structural context

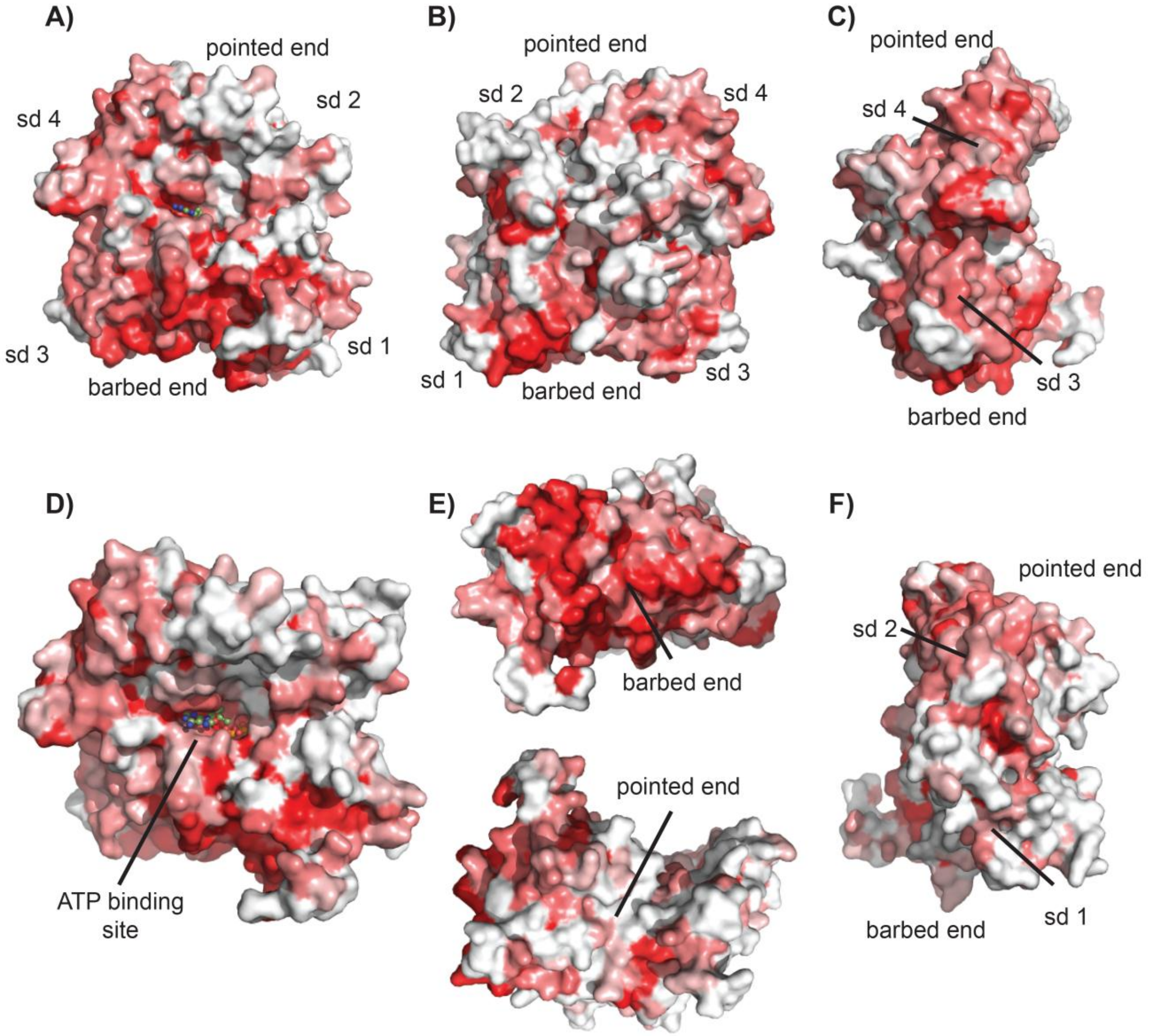

Supplementary Figure S4: Amino acid sequence conservation of human Arp8.
The molecular surface representations of human Arp8 colored according to amino acid conservation scores calculated from multiple sequence alignments of human Arp8 by the ConSurf web server (http://consurf.tau.ac.il): from red (absolutely conserved) to white (not conserved). (A) Human Arp8 in the classical view of the actin fold and rotated by 180° (B). (C) Side view on subdomains (sd) 3 and 4 (inner domain) show that they are more conserved than the side of subdomains 1 and 2 (F). (D) The accessible ATP binding site is highly conserved. (E) The barbed end and especially the target-binding cleft of Arp8 are more conserved than the pointed end. (F) Side view on subdomains 1 and 2 (outer domain) show less conservation compared to the inner domain (C).

ATPase activity of Arps and subcomplex I

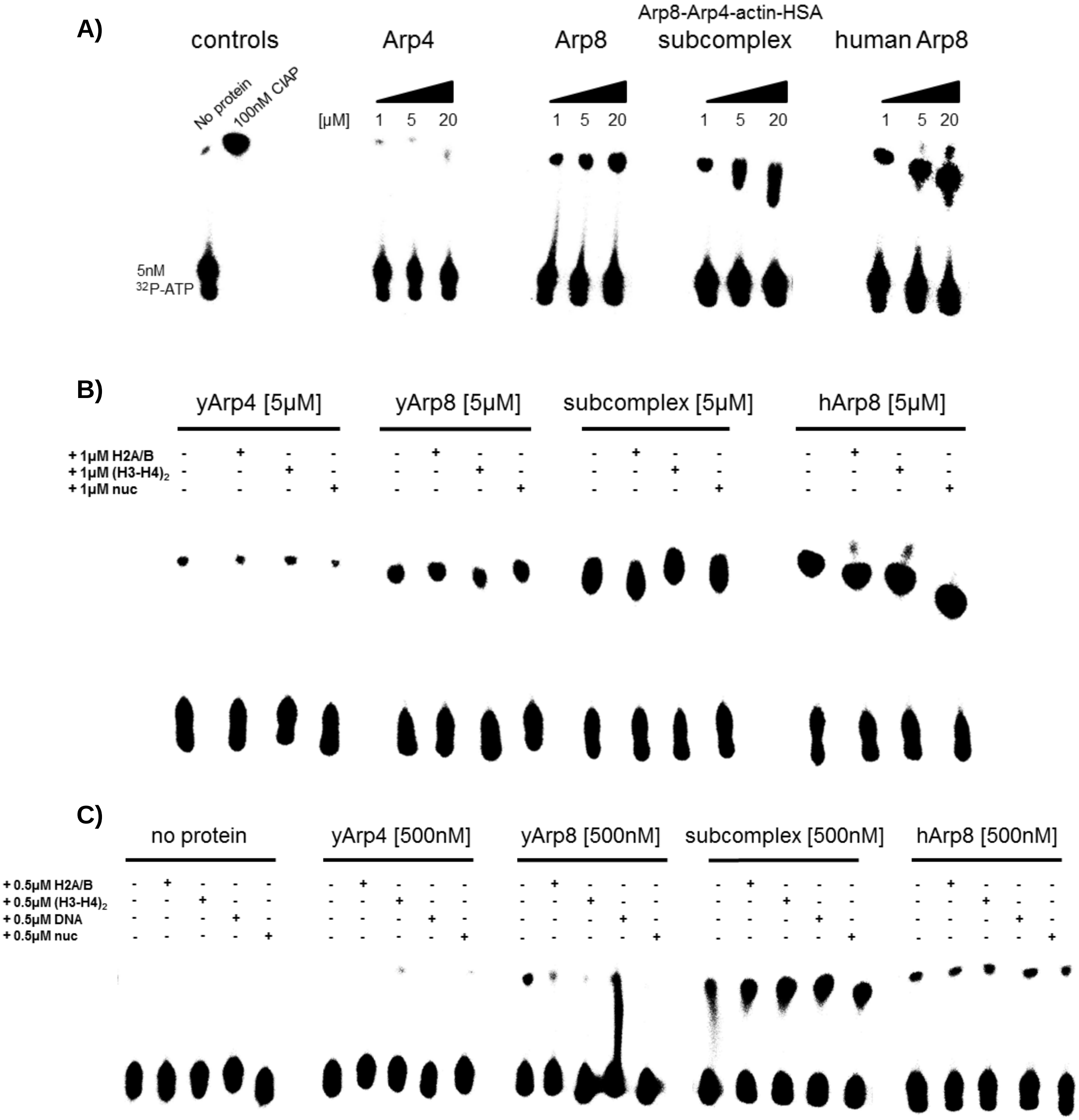

Supplementary Figure S5: Basal [γ-^{32}P] ATP hydrolysis by Arps and lack of ATPase stimulation by nucleosomes.
Chromatography of the ATPase reaction sample (incubated for 4 hours at 30°C) separates [γ-^{32}P] ATP from hydrolyzed [γ-^{32}P] phosphate. (A) Basal activity of ATP hydrolysis by Arps in high concentrations. (B,C) No significant stimulation of the ATPase activity could be observed in presence of H2A-H2B, $(H3\text{-}H4)_2$ or nucleosomes.

We observed a low basal ATPase activity for yeast and human Arp8 as well as the subcomplex Arp8-Arp4-actin-HSA but not for Arp4, which is in line with the structure-based analysis of the respective active sites. As we also quantified the binding of the Arps to nucleosomes and constituents thereof, we probed for Arp ATP hydrolysis in presence of these binding partners. However, no significant stimulation of the ATPase activity could be measured. Due to the high concentrations of Arp8 and the subcomplex needed to visualize ATP hydrolysis, we could not add binding partners in excess. Nonetheless, even if activation could only partially occur, we would have expected a significant stimulation in the hydrolysis rate of ATP, which is not the case.

Crystal contacts of human Arp8

We noted that symmetric crystal contacts between N-terminal and C-terminal residues of two adjacent Arp8 molecules might shed light on a possible interaction between two human Arp8 proteins. For further analysis, the structure was submitted to the PDBe server PISA (Protein Interfaces, Surfaces and Assemblies) (56). The complex formation significance score (CSS) was calculated to be 0.186, which indicates that the interface can play an auxiliary role in complex formation. The interface area comprises 1126 $Å^2$ and involves 38 amino acids of each monomer. Six hydrogen bonds (distances < 3.25 Å) are involved and contribute to a calculated interface $\Delta^i G$ of -15.6 kcal/mol. The missing N-terminal amino acids (1-33) in the crystallized construct comprise a characteristic sequence motif of alternating lysine and glutamate residues, which might add several more contacts for a possible human Arp8 dimerization. However, we could not observe any dimerization in solution for purified full-length yeast or human Arp8 (Supplementary Table S1). Therefore, the contacts made in the crystal lattice do not occur or do at least not suffice for Arp8 dimerization in solution but could indeed play a role in a putative Arp8 dimerization triggered by auxiliary factors, e.g. at its nucleosome substrate.

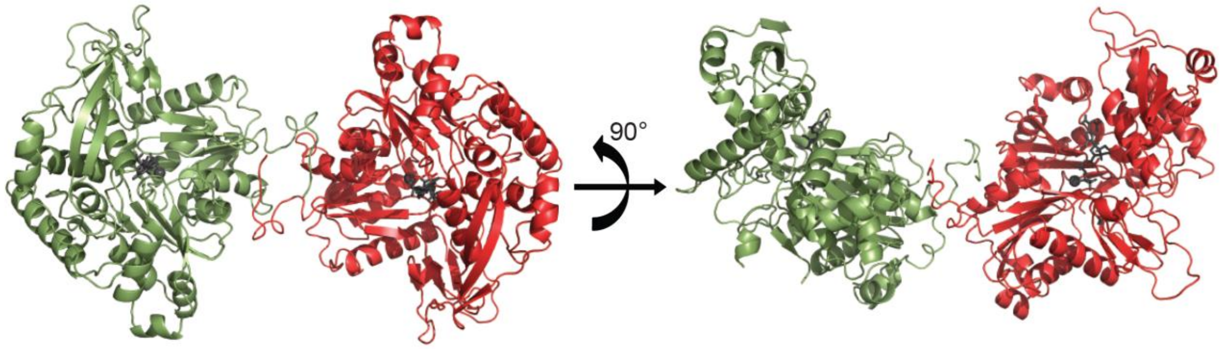

Supplementary Figure S6: Dimer of human Arp8 formed in the crystal.
Two human Arp8 (Δ1-33) molecules (green and red) contact each other at the truncated N-terminus and C-terminal residues. The mostly hydrophobic surface area comprises 1126 $Å^2$ and could serve as an auxiliary interaction surface in dimer formation.

Small angle X-ray scattering (SAXS) of human Arp8

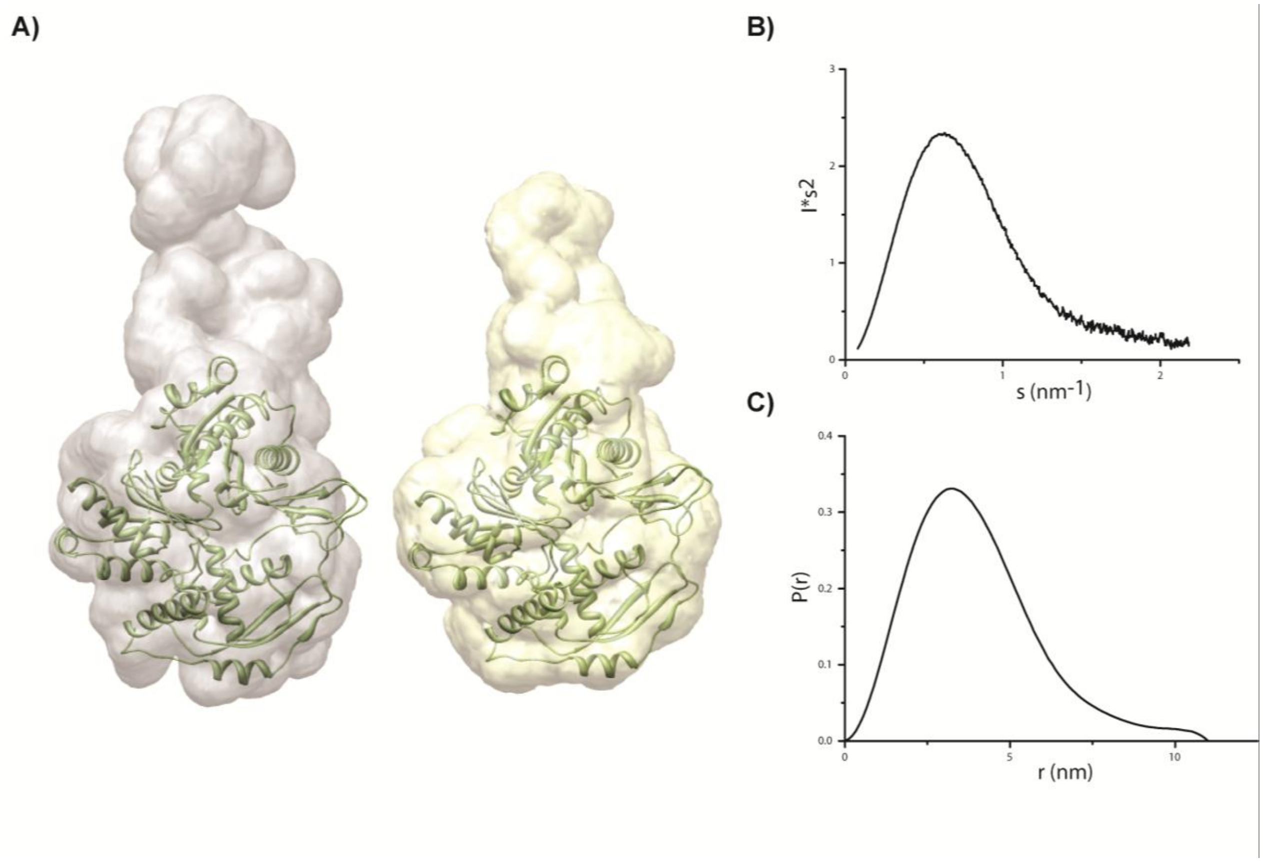

Supplementary Figure S7: Small Angle X-ray Scattering (SAXS) structure of human Arp8.
(A) Comparison of SAXS GASBOR *ab initio* structure calculations for full-length (grey) and the N-terminally truncated human Arp8 (orange) with the crystal structure of human Arp8 Δ1-33 (green) docked into the SAXS density. The protrusion is slightly elongated in the full-length protein, but otherwise the SAXS structures are virtually identical. Insertion IV of the Arp8 structure, which is missing in the atomic resolution model, can be allocated to the convex part of the actin fold and mostly the protrusion. (B) The Kratky-plot (I*s² *vs.* s) of the truncated hArp8 shows a bell-shaped curve typical of a folded protein. (C) P_r distribution function shows a slight tailing to higher radii indicating a potential elongated protrusion as also seen in the *ab initio* models (A).

Molecular weight of Arp4, Arp8 and subcomplex I derived from static light scattering (SLS) and SAXS

Supplementary Table S1: Molecular weight of Arp4, Arp8 and the conserved INO80 subcomplex I determined by a combination of size exclusion chromatography (SEC) with static light scattering (SLS) and small angle X-ray scattering (SAXS). n.d. = not determined.

Sample	Calculated monomeric molecular weight [kDa]	Molecular weight determined by SEC/SLS [kDa]	Polydispersity [M_W/M_N]	Molecular weight derived from Porod-volume (SAXS) [kDa]
human Arp8 (full-length)	70.5	70	1.001	78
human Arp8 (Δ1-33)	66.9	69	1.006	70
yeast Arp8	100.2	101	1.001	105
yeast Arp4	54.8	56	1.004	51
Yeast subcomplex I (Arp8-Arp4-actin-HSA)	223.2	220	1.003	n.d.
BSA (calibration control)	66.5	68	1.003	n.d.

Molecular weight determination by SEC/SLS was performed using an ÄKTAmicro system with a Superdex 200 15/150 GL column (GE Healthcare) connected to a static light scattering / refractive index measurement device (Viscotek TDA270). For measurements, the supernatant after centrifugation (15 min., 16100 rcf, 4°C) was used. Calibration of the system was done with a sample of BSA (66.5 kDa) prior to the measurements of the samples. An independent BSA run at the end was added to confirm calibration and stability of the system. Data evaluation was performed using the OmniSEC software package. Curve runs of the calculated molecular weight for the analyzed peaks were all linear indicating a single molecular weight species in the respective peak, as e.g. shown for full-length human Arp8 in Supplementary Figure S8.

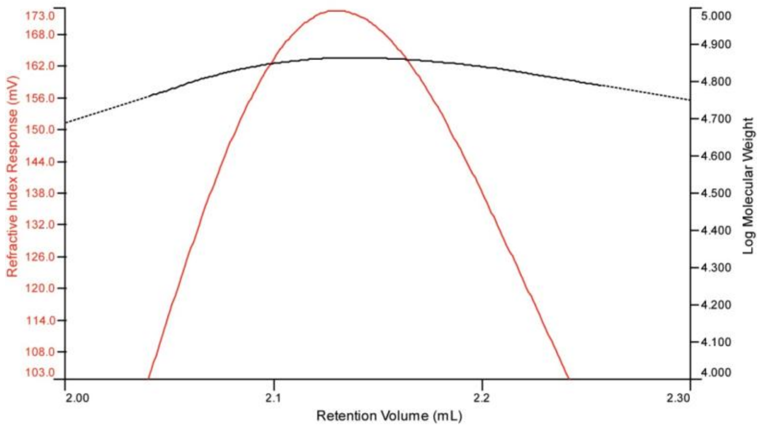

Supplementary Figure S8: Monodispersity of SLS samples.
Curve run of the calculated log (molecular weight) of full-length human Arp8 shown in black with the refractive index curve shown in red as a representative sample. The almost horizontal molecular weight curve within the limits of the evaluated peak indicates a single monodisperse molecular weight species (polydispersity M_W/M_N=1.001).

DNA binding of Arps shown by EMSA and fluorescence titration

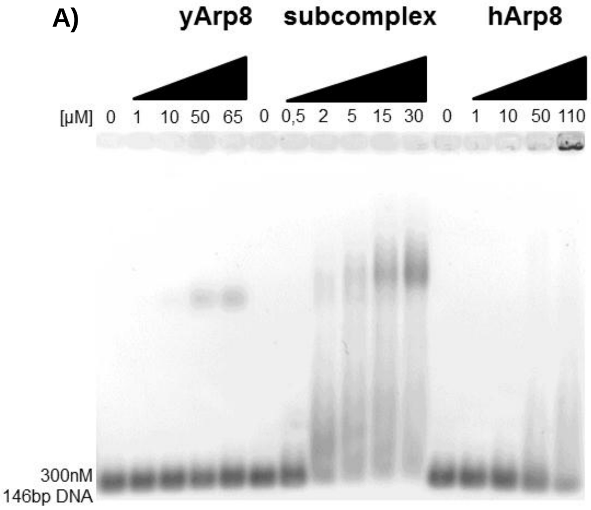

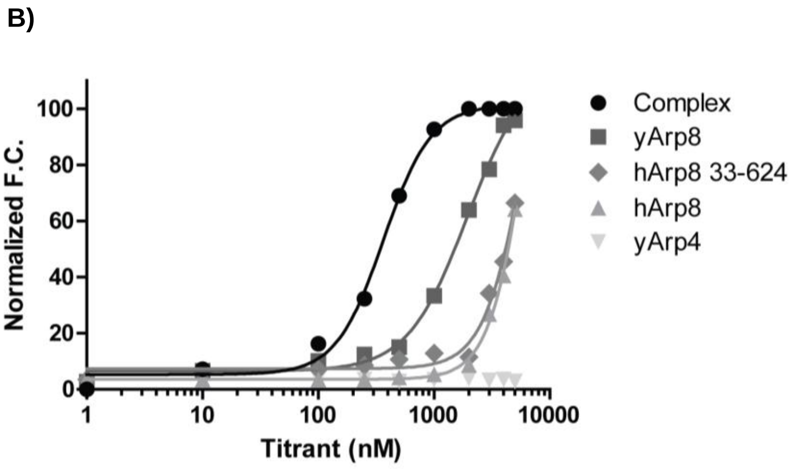

Supplementary Figure S9: DNA binding of actin-related proteins.
(A) Electrophoretic mobility shift assays and (B) fluorescence titration curves both show weak DNA binding for yeast and human Arp8 in the micromolar range and DNA binding of the subcomplex in a high nanomolar range. Therefore, nucleosome binding of the INO80 subcomplex (Arp8-Arp4-actin-HSA) is likely to involve DNA binding by the HSA domain. Arp4 had no measurable affinity to DNA in our assays.

Supplemental References

55. Larkin, M.A., Blackshields, G., Brown, N.P., Chenna, R., McGettigan, P.A., McWilliam, H., Valentin, F., Wallace, I.M., Wilm, A., Lopez, R. *et al.* (2007) Clustal W and Clustal X version 2.0. *Bioinformatics*, **23**, 2947-2948.

56. Krissinel, E. (2010) Crystal contacts as nature's docking solutions. *Journal of Computational Chemistry*, **31**, 133-143.
